# Evaluation of the Accuracy of a Fused Deposition Modeling Process in the Production of Low-Density ABS Lattice Structures

**DOI:** 10.3390/ma18071679

**Published:** 2025-04-07

**Authors:** Gianluca Parodo, Luca Sorrentino, Sandro Turchetta, Giuseppe Moffa

**Affiliations:** Department of Civil and Mechanical Engineering, University of Cassino and Southern Lazio, 03043 Cassino, Italy; sorrentino@unicas.it (L.S.); turchetta@unicas.it (S.T.); giuseppe.moffa@unicas.it (G.M.)

**Keywords:** ABS, fused deposition modeling, lattice structures, geometrical/dimensional accuracy

## Abstract

Fused Deposition Modeling (FDM) has emerged as one of the most widely adopted additive manufacturing (AM) technologies due to its broad material availability and low production costs, enabling the efficient production of complex geometries and customized components. Among the materials commonly used in AM, Acrylonitrile Butadiene Styrene (ABS) is particularly notable for its favorable mechanical properties, ease of processing, and versatility. While moderate-to-high-density lattice configurations have been extensively studied, low relative density lattice structures remain largely unexplored. This study investigates the feasibility of fabricating Cuboidal Body-Centered Cubic (BCC) lattice structures with relative densities of 5%, 10%, and 15% using FDM. The geometrical/dimensional accuracy of the printed samples is thoroughly assessed to quantify fabrication-induced deviations, focusing on strut geometry and overall lattice consistency. Results show that while smaller lattice configurations, particularly those with 5% relative density, exhibit significant geometrical inaccuracies due to printing limitations (e.g., strut waviness, material deposition inconsistencies, layer misalignment), larger configurations demonstrate improved dimensional and geometrical fidelity and structural integrity. A framework is proposed for assessing geometrical/dimensional fidelity, which can enhance the predictive modeling of these structures and optimize manufacturing processes. These findings clarify low relative density lattice manufacturability, guiding research on mechanical performance for lightweight aerospace applications.

## 1. Introduction

Lattice cell structures (LCSs) are widely employed in applications that demand lightweight, high strength, and energy absorption, particularly in the aerospace, automotive, and biomedical fields. By leveraging their repeating unit cell patterns, LCSs significantly reduce weight and material consumption compared to solid parts. Their geometry can be tailored to meet specific mechanical requirements, such as stiffness and energy absorption, with various configurations designed to enhance buckling resistance and energy absorption efficiency. Due to these characteristics, LCSs have been extensively studied as core materials for sandwich panels in aerospace structures, where they can offer an optimal balance between mechanical performance and weight reduction [1,2].

The adoption of lattice structures in the aerospace and industrial sectors is currently limited by high costs and manufacturing complexity. Advanced additive technologies, such as Selective Laser Sintering (SLS), ensure high geometrical quality and superior mechanical properties, but their use is often economically prohibitive for large-volume components. Conversely, Fused Deposition Modeling (FDM) could represent a low-cost alternative that could make the production of lattice structures more accessible, particularly as substitutes for traditional polymer foams used in sandwich panels. However, FDM has intrinsic limitations related to printing resolution, material deposition, and the geometrical stability of thin elements. Consequently, it is crucial to assess to what extent FDM technology can ensure sufficient dimensional and geometrical accuracy for its use in aerospace structural components, identifying potential constraints that may limit its manufacturability.

ABS, a widely utilized engineering thermoplastic polymer, is also one of the most commonly employed materials in FDM. Its composition includes over 40% styrene, which provides rigidity and processability; acrylonitrile, which enhances chemical resistance and thermal stability; and butadiene, which contributes toughness and impact strength [3,4,5]. ABS lattice structures have been used in lightweight components in the aerospace industry due to their high strength-to-weight ratios. In biomedical applications, they have been used due to their biocompatibility and factors such as layer adhesion, print orientation, and infill density.

The mechanical behavior of ABS lattice structures fabricated via FDM has been widely investigated, providing valuable insights into their bending, compression, and overall structural integrity. However, inherent material characteristics of ABS, such as thermal expansion and shrinkage, induce geometrical deviations both during and after the printing process. These deviations primarily result from the semi-molten state of ABS during extrusion, which leads to dimensional instability as the material undergoes cooling and solidification [6]. To improve stiffness and thermal stability, carbon fiber reinforcement is commonly integrated into ABS. While this reinforcement enhances mechanical performance, it also introduces complexities in dimensional precision due to the anisotropic behavior introduced by the deposition process [7].

Research has emphasized the pivotal role of lattice designs in optimizing performance. Design parameters, including process-induced anisotropy and infill architecture, require careful consideration. The layer-by-layer deposition characteristic of FDM introduces anisotropic behavior, resulting in geometrical distortions, particularly in complex lattice structures. However, design strategies that account for these anisotropic effects, such as density-variable topology optimization, can mitigate these deviations [8]. Additionally, infill architectures—such as grid, hexagonal, or triangular configurations—significantly impact both dimensional stability and mechanical performance. Among these, hexagonal patterns often provide an optimal balance in stress distribution, enhancing overall structural efficiency [7].

The simple cubic (SC) lattice, as described by Libonati et al. [9], serves as a fundamental framework structure for mechanical testing and analysis, while the BCC lattice, studied by Ali and Abdi [10], is widely studied for its energy absorption capabilities. They optimized this structure using multi-objective genetic algorithms to enhance stiffness while minimizing stress under load. Libonati et al. [9] also noted that the Face-Centered Cubic (FCC) lattice exhibits structural efficiency and is often employed in applications requiring lightweight materials with high strength. Arañez et al. [11] and Maconachie et al. [12] highlighted the excellent mechanical properties of Gyroid lattice structures, including high tensile strength and energy absorption capabilities. Furthermore, Arañez et al. [11] demonstrated how these structures can be designed with varying material distributions to enhance performance under different loading conditions.

Shamim et al. [13] have demonstrated that different lattice geometries result in varying mechanical properties. Tetrahedron-cubic lattice design has been identified as particularly effective in load-bearing applications, exhibiting superior bending rigidity (15.36 N/mm) and energy absorption capacity (38.54 J/g) compared to alternative lattice configurations. Similarly, Ghosh et al. [14] demonstrated that incorporating plates into simple cubic and body-centered cubic lattices significantly enhances compressive performance, with modulus improvements between 125% and 393% and energy absorption increases ranging from 17% to 395% compared to traditional open lattice structures. Bouteldja et al. [15] revealed that ABS lattice structures exhibit a notable increase in compressive strength under dynamic loading conditions, demonstrating strain rate sensitivity.

Poddar et al. [16] explored the potential of advanced manufacturing techniques, such as axial lattice extrusion, to significantly enhance the compressive strength of ABS composites, achieving impressive values of around 17.4 MPa. The use of functionally graded materials and advanced manufacturing techniques, such as axial lattice extrusion, according to the study, improves the mechanical properties of ABS structures by optimizing fiber alignment and reducing polymer interfaces.

Further studies, such as those by Monkova et al. [17], have evaluated the impact of relative density (also named volume fraction or specific volume) and cell topology on the tensile properties of various lattice structures. Cartesian, Octagonal, Starlit, and Rhomboid structures demonstrated unique behaviors under tensile stress, with the Cartesian structure achieving superior mechanical properties. The Rhomboid structure, despite its lower tensile strength, excelled in energy absorption and toughness, making it suitable for applications involving impact stresses. In bending studies, Monkova et al. [18] found that the Starry structure exhibited the best overall bending properties, combining stiffness, energy absorption, and strength across tested relative densities.

The performance of lattice cell structures can also be enhanced through advanced manufacturing techniques. Alwattar et al. [19] compared traditional BCC lattice cell structures with InsideBCC (named also as Cuboidal BCC) composite lattice designs, demonstrating that the addition of vertical and horizontal struts significantly improves stiffness, failure load, and energy absorption. Similar findings have been reported by other researchers who examined the effect of vertical struts on BCC lattice cell configurations [20,21,22]. These improvements underscore the importance of optimizing strut arrangement and geometrical parameters to enhance mechanical behavior.

Despite the extensive study of ABS lattice structures, most research focuses on moderate-to-high-density configurations, which provide an optimal balance between mechanical strength and weight. However, in aerospace applications, where mass reduction is a top priority, ultra-low-density structures could serve as an effective core material for sandwich panels, potentially replacing traditional polymer foams. Despite their potential, the existing literature provides little information on the accuracy of these structures when manufactured using FDM, a process inherently limited by material deposition constraints and printing resolution. Moreover, FDM is rarely employed for fabricating core components; its primary advantage lies in its economic feasibility for prototyping and low-cost production. Indeed, while higher-resolution techniques such as SLS and SLA are commonly used for complex-shaped components [23,24], different studies have highlighted that FDM remains a viable alternative when economic considerations are paramount [25,26]. Dimensional and geometrical deviations in the struts can significantly affect the mechanical performance of low relative density lattice structures, altering their specific strength and energy absorption capacity.

This study aims to assess the manufacturability of low relative density Cuboidal BCC lattice structures fabricated via FDM, emphasizing their dimensional precision and overall geometric characteristics. The geometrical and dimensional accuracy of both lattice cells and individual struts is investigated through microscopic analysis, identifying fabrication-induced deviations. Particular attention is given to the limitations of the FDM process in accurately replicating fine strut features, considering the constraints imposed by its technological signature. Geometrical/dimensional deviations are quantified to evaluate the accuracy of the FDM process in the production of low-density ABS lattice cells, offering insights into their suitability as lightweight core materials in aerospace and structural engineering applications.

## 2. Materials and Methods

This section provides a detailed overview of the materials and methods employed in the investigation. It outlines the design and fabrication process of the structured specimens using FDM with ABS filament. Particular attention is given to the characterization of the specimens, including dimensional measurements and the evaluation of the geometrical accuracy.

The relative density percentage (M) can be expressed as (1).

(1) $$M=\frac{\rho_{latiice}}{\rho_{solid}}\times 100$$

where ρ_lattice_ and ρ_solid_ denote the density of the lattice and the density of the solid material constituting the lattice structure, respectively.

**Figure 1:**
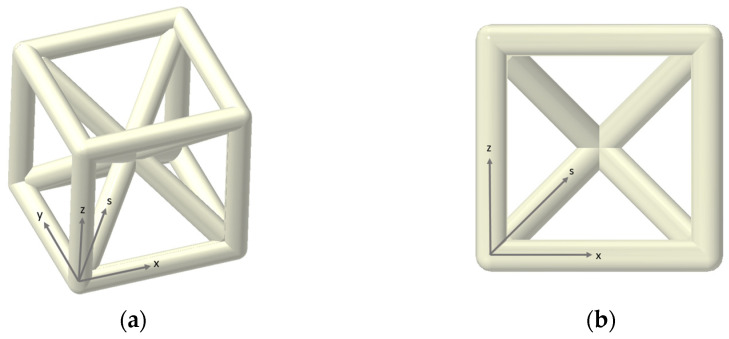
A schematic illustration of the Cuboidal Body-Centered Cubic (BCC) lattice structures, showing the isometric view (**a**) and the front view (**b**), with a reference system indicating the strut orientation.

To systematically describe the orientation of the lattice struts, a reference system was defined, as shown in Figure 1. The x and y directions correspond to struts parallel to the printing orientation, while the z direction represents struts orthogonal to the printing orientation. Additionally, the s direction is introduced to denote struts inclined with respect to the printing orientation.

### 2.1. Fabrication of ABS Lattice Cells

ABS Cuboidal BCC lattice structures were fabricated using FDM technology. ABS filament with a nominal diameter of 1.75 mm was used. This filament is recognized for its balanced mechanical properties and ease of processing, offering tensile strength in the range of 30 ÷ 50 MPa.

Printing was performed using a Stratasys F190 CR printer. The process parameters defined by Stratasys to optimize layer adhesion and minimize residual stresses were applied without modification in this study, as they are fixed by the manufacturer and not adjustable by the user. The manufacturer-specified settings are presented in Table 1.

These parameters align with the study’s objective of evaluating FDM printing performance under constrained conditions, specifically assessing the printer’s capabilities within a predefined operational framework. Notably, this research addresses a gap in the existing literature by examining the manufacturability and dimensional and geometrical accuracy of low relative density BCC lattice structures produced by FDM.

The study focused on lattice structures with three relative densities “M” (5%, 10%, and 15%) and lattice cell dimensions “N” of 5 mm, 10 mm, 15, and 20 mm. The configurations are detailed in Table 2.

During fabrication, the lattice’s complex geometry, including overhanging struts and nodal connections, necessitated the use of water-soluble support material to maintain structural integrity. The supports were printed with maximum allowable filling density to enhance rigidity and reduce defects such as layer misalignment. The support material used was QSR (Quick Soluble Release), a proprietary water-soluble material commonly employed with Stratasys systems. After printing, the support material was dissolved in a mild water-based solution, consisting of water and a gentle sodium hydroxide (NaOH)-based solution, ensuring that the lattice cells were free of artifacts.

### 2.2. Quality Control of Printed Lattice Cells and Strut Diameter Characterization

This section presents the quality control measures adopted to ensure the fabricated lattice structures closely matched the nominal geometry and design specifications. The evaluation first focused on the dimensional and geometrical accuracy of the printed lattices, assessing deviations from the nominal design to quantify manufacturing precision. Following this, the strut diameter characterization was detailed, as it is critical for understanding the structural integrity and mechanical response of the lattices. This analysis quantified the average strut diameter and its variation with respect to the printing direction, providing insights into process-induced discrepancies. Finally, weight cell analysis was conducted, where the measured weight of the fabricated structures was compared against theoretical values to assess material deposition accuracy. This evaluation also served as an indirect measure of porosity, offering insights into the presence of voids or inconsistencies within the printed material, which may influence the mechanical performance of the lattice structures. By integrating these assessments, a comprehensive understanding of both geometric and dimensional accuracy, and material distribution was achieved, contributing to the overall reliability of the printed lattice structures.

#### 2.2.1. Geometric Assessment and Dimensional Accuracy of Printed Lattice Cells

To evaluate the geometric and dimensional accuracy of the fabricated lattice structures, an optical microscopy analysis was conducted using a Leica Ivesta 3. The primary objective was to assess deviations from the nominal geometry and quantify manufacturing precision. High-resolution images were captured and processed using an image analysis software, enabling detailed measurements of the unit cell edge dimensions. These measurements were performed manually at 0.5 mm intervals along the x, y, and z directions, ensuring a comprehensive assessment of the dimensional consistency. This methodology directly accounted for variations, defects, and irregularities introduced during the printing process, providing an accurate representation of the fabricated structures.

#### 2.2.2. Strut Diameter Characterization

Strut diameter characterization was performed to analyze the dimensional consistency of the individual structural elements forming the lattice. The analysis considered the average strut diameter and its variation relative to the printing direction, as these parameters critically influence the mechanical performance of the structure. To ensure statistical reliability, measurements were conducted on five specimens per lattice configuration and across all principal orientations (x, y, z, and s directions), as defined in Figure 1. Struts were carefully sectioned and positioned to lie in a planar orientation, improving measurement precision and ensuring proper focal alignment during microscopy examination. Image analysis software was used to determine cross-sectional dimensions at a spacing of 0.2 mm along the strut axis. Additionally, surface quality was assessed to identify potential irregularities induced by the additive manufacturing process. This characterization provided valuable insights into dimensional variations and process-induced inconsistencies, allowing for a comprehensive understanding of the printed struts.

#### 2.2.3. Weight Cell Analysis

A weight analysis was conducted to evaluate material deposition accuracy and identify potential porosity within the printed structures. Each sample was weighed using a KERN ADB 200-4 high-precision analytical balance, ensuring reliable and repeatable measurements. To account for process variability, five specimens per lattice configuration were manufactured and measured, and the mean weight and standard deviation were calculated to assess fabrication consistency.

The nominal weight of each configuration was determined using the corresponding CAD model, where the material density provided by the ABS manufacturer (*ρ* = 1.05 g/cm^3^) was assigned. Since this theoretical weight assumes a fully dense material without voids, discrepancies between the measured and nominal weight can be attributed to porosity, internal voids, or printing inconsistencies.

The weight relative error (ε_Wn-Wr_) was determined by comparing the measured weight of the specimens to their nominal weight (2).

(2) $$\varepsilon_{Wn-Wr}=\frac{W_{n}-W_{r}}{W_{n}}\times 100$$

To further quantify the impact of porosity on the lattice structure, an effective weighted diameter (d_n,ew_) was derived to account for the void presence and non-uniform material distribution along the lattice struts. This parameter was obtained by iteratively adjusting the strut diameter within the CAD model until the weight calculated from the CAD—multiplying the volume derived through the CAD model to the ABS material density provided by the manufacturer—matched the experimentally measured weight. In other words, the iterative process continued until the weight error, defined as the difference between the computed and measured weight, was reduced to zero. As a result, d_n,ew_ provides a simplified yet accurate representation of the lattice structure by incorporating the effects of porosity and the variability in material distribution along the struts. The analysis was conducted under the assumption of uniform strut geometry, implying that the struts exhibited a consistent diameter along their length and were devoid of printing defects or material inconsistencies. This assumption enabled an indirect yet quantitative evaluation of the effects of porosity.

To further assess the discrepancy between the nominal strut diameter (d_n_) and the effective weighted diameter (d_n,ew_), the relative error (ε_dn-dn,ew_) was calculated (3)

(3) $$\varepsilon_{dn-dn,ew}=\frac{d_{n}-d_{n,ew}}{d_{n}}\times 100$$

By integrating this weight-based analysis with the dimensional characterization, a more comprehensive understanding of material distribution and process-induced variations was obtained.

## 3. Results and Discussion

### 3.1. Fabrication of Lattice Cells

**Figure 2:**
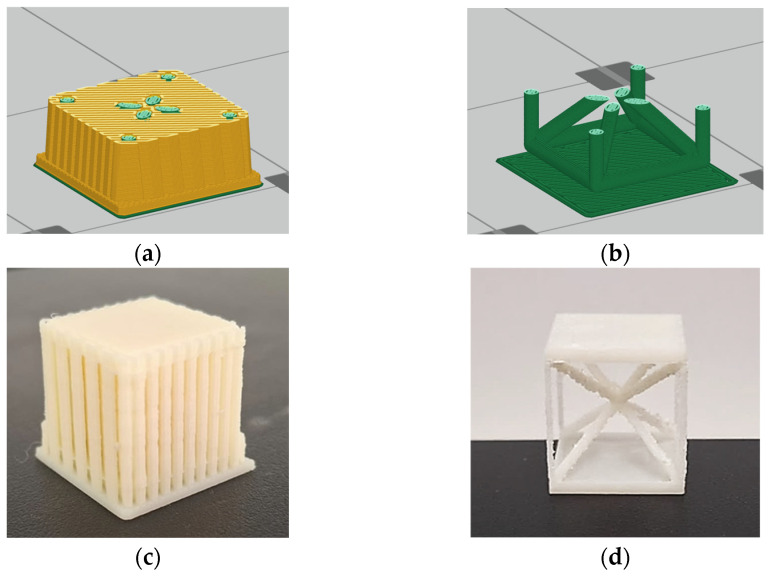
Illustration of the slicing model with support material visualized (**a**), the slicing model with support material hidden (**b**), the as-fabricated geometry with support material still in place (**c**), and the final cell structure after the support material dissolved (**d**).

As outlined, ABS Cuboidal BCC lattice cells were printed using the maximum allowable filling density of the support material to ensure the structural integrity of the printed cells. A representation of a lattice cell post-printing, with the support material intact, is shown in Figure 2c,d. Following the printing phase, the support material was selectively dissolved through a chemical treatment, which removed the support material without affecting the integrity of the lattice structure. This step was crucial to ensure the lattice cells exhibited clean and precise geometries, free from residual interference from the support material.

**Figure 3:**
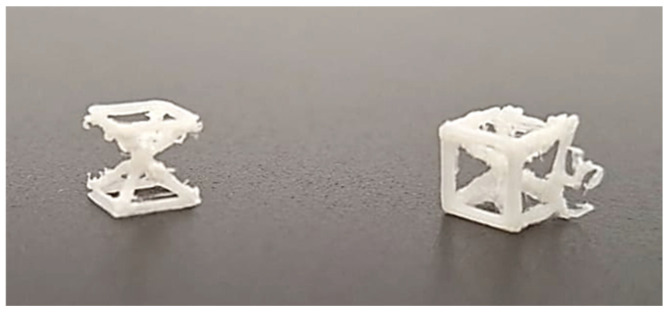
Damaged 5 mm lattice cell specimen (M = 10%, **left**; M = 15%, **right**) following printing process, highlighting structural failure due to material or process constraints.

Despite significant efforts to optimize the manufacturing process, certain dimensional configurations posed considerable challenges. Lattice cells with a nominal dimension of 5 mm exhibited critical fabrication issues. The small size of these cells led to fragile struts that frequently fractured during or immediately after the printing process, rendering them unsuitable for further analysis (Figure 3). This failure highlighted the limitations of the FDM process in reliably producing lattice cells with very small dimensions, particularly when using ABS filament. Consequently, all 5 mm cells were excluded from subsequent evaluations.

### 3.2. Quality of ABS Lattice Cells

To assess the accuracy of the FDM process in the production of low-density ABS lattice cells, a comprehensive characterization was performed, encompassing geometric, dimensional, and weight analyses. The objective was to quantify deviations from the nominal design and evaluate the reliability of the fabrication process in producing structurally consistent lattice elements.

#### 3.2.1. Geometric Assessment and Dimensional Accuracy of Printed Lattice Cells

**Figure 4:**
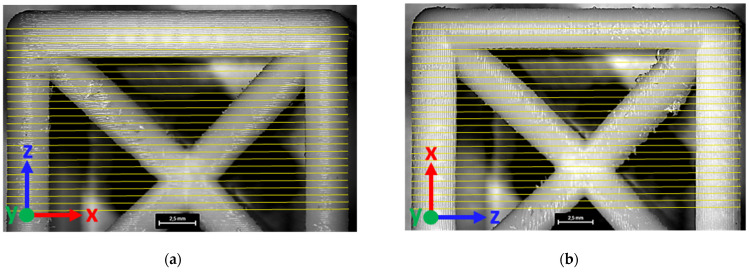
Unit cell dimensions measured using image processing techniques, with measurement spacing of 0.5 mm along x, y (**a**), and z (**b**) printing directions, respectively. Measurements were taken separately for each direction due to magnification limitations of microscopy.

Optical microscopy analysis provided further insights into the geometric accuracy of the printed lattice cells. Image processing techniques (Figure 4) enabled precise measurement of the unit cell edge dimensions, allowing for a direct comparison with the nominal values.

The dimensional variations in the manufactured lattice structures, ranging from 0.6% to 1.8% relative to the nominal values, were calculated by comparing the measured unit cell edge dimensions with the nominal values. The deviation for each lattice configuration was calculated (4). These variations did not exhibit a consistent correlation with any specific lattice parameter, such as cell size, relative density, or nominal strut diameter.

(4) $$\delta =\frac{\left|N_{r}-N_{n}\right|}{N_{n}}\times 100$$

where N_n_ and N_r_ represent the nominal and measured cell edge dimensions, respectively.

**Figure 5:**
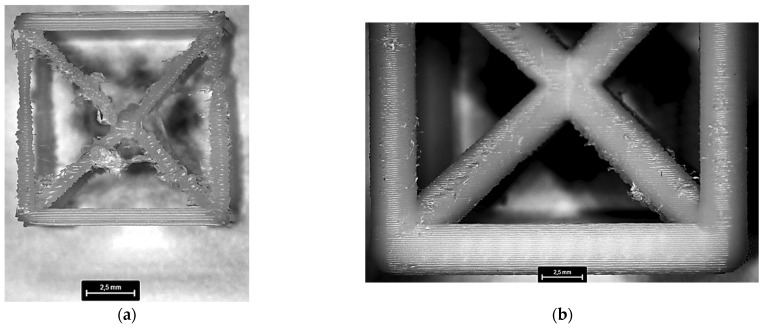
Comparison of printed lattice structures highlighting localized defects. L10_Dr5 configuration (smallest cell dimension and lowest relative density) exhibits noticeable strut waviness and filament deposition inconsistencies (**a**), whereas L15_Dr15 configuration (largest cell dimension and highest relative density) demonstrates improved geometric and dimensional accuracy (**b**).

Furthermore, localized defects were identified, particularly in configurations with smaller cell dimensions and lower relative densities (Figure 5). These defects primarily manifested as strut waviness and inconsistencies in filament deposition, likely resulting from limitations in the printing process and insufficient structural support during fabrication. This occurred despite employing the maximum infill density for the support material.

#### 3.2.2. Analysis of Strut Dimensional Accuracy

**Figure 6:**
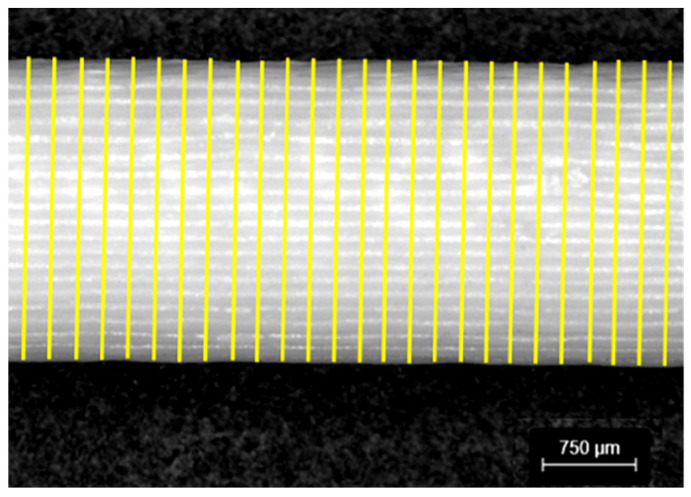
Strut diameter measurements using image processing technique with measurement spacing of 0.2 mm along strut axis.

Following the procedures outlined previously, the strut dimensions were systematically assessed using microscopy and image processing technique (Figure 6) to identify localized deviations of the printed lattice structure from the nominal design. This method allowed for a comprehensive analysis of strut dimensional accuracy across various configurations and printing orientations, contributing to a deeper understanding of the lattice’s geometric fidelity.

**Figure 7:**
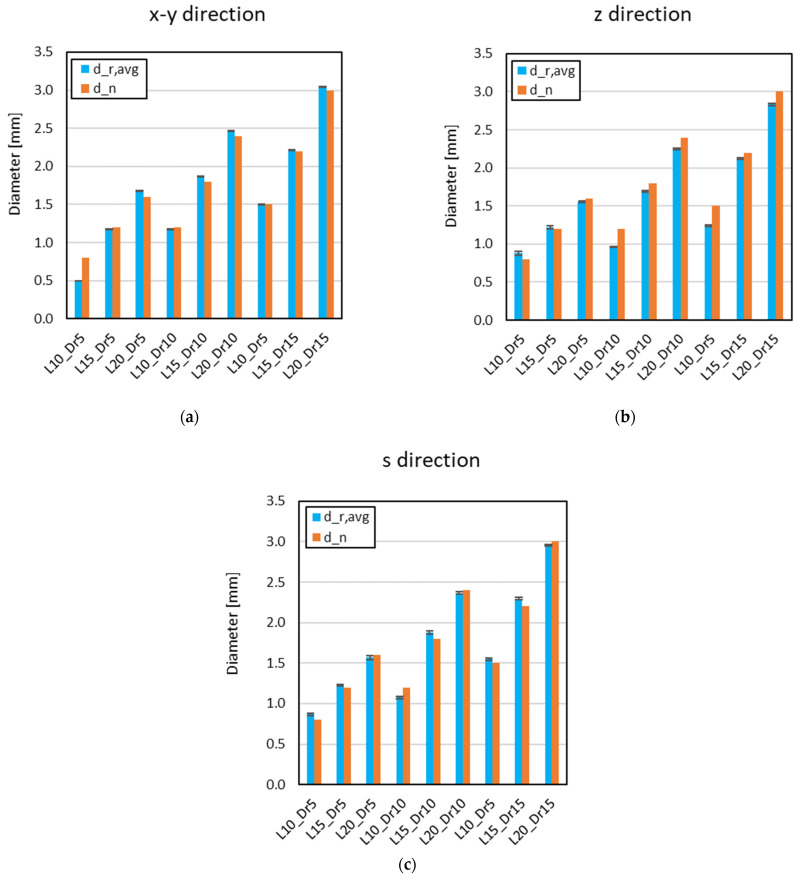
Relationship between d_n_ and d_r,avg_, as function of orientation relative to printing direction: x and y directions (**a**), z direction (**b**), and s direction (**c**) for cell configurations investigated.

Figure 7 presents the relationship between the nominal strut diameter (d_n_) and average strut diameter (d_r,avg_), across various orientations relative to the printing direction and for the different cell configurations investigated. The extensive dataset gathered from multiple samples provided a thorough evaluation of dimensional variations, ensuring a robust comparison between the nominal design and the printed structures.

The analysis of dimensional variations revealed that parallel-oriented struts exhibited the highest geometric accuracy, with the smallest deviations between the nominal and measured values. This can be attributed to the layer-by-layer deposition process, where struts aligned with the printing direction benefit from precise extrusion and reduced geometric inconsistencies in the outer material layer. However, in certain configurations (in particular, L10_Dr5), the average measured diameter deviated significantly from the nominal value. This discrepancy arises from the slicing algorithm’s optimization process, which determines whether to deposit an additional layer or not. Depending on this decision, the final strut diameter may be overestimated or underestimated, as the printer adjusts to maintain structural continuity, occasionally leading to local material accumulation or insufficient deposition. These results highlight the influence of printing direction on dimensional accuracy, providing insights into potential sources of variation in the fabrication process.

To analyze the geometrical and dimensional characteristics of the cell configurations, the average strut diameters across all orientations were calculated (mean d_r,avg_). This involved averaging the strut diameters for each orientation within the specific cell configuration. This approach provides a representative measure of the strut dimensions, accounting for variability and minimizing the influence of orientation-specific effects.

The calculated average diameter was then compared with the nominal diameter, demonstrating satisfactory correlation (Table 3).

#### 3.2.3. Weight Cell Analysis

While microscopy analysis provided detailed insights into the geometrical and dimensional features of the cell configurations, they alone were insufficient for a comprehensive evaluation of the structure accuracy. This is because microscopy primarily focuses on visual dimensions, which may not capture all variations in the material distribution. To fully assess the geometric and dimensional accuracy, weight measurements were also necessary. This provided additional data on the uniformity and precision of the manufacturing process, enabling a more robust assessment of the lattice accuracy.

**Figure 8:**
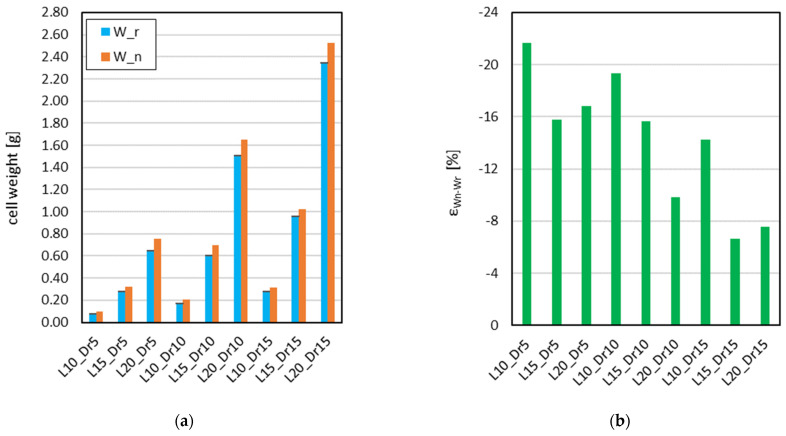
Cell weight distribution, for the cell configurations analyzed, compared to their respective nominal values (**a**), and the corresponding weight relative error “ε_Wn-Wr_” (**b**).

The weight measurements of the printed lattice cells (W_r_) were compared with the theoretical weight obtained from the CAD model (W_n_), assuming ideal deposition conditions. In Figure 8, a comparison is illustrated between W_n_ and W_r_.

The measured weight values exhibited variations across the five specimens analyzed for each lattice configuration. The mean weight and standard deviation were computed to assess fabrication consistency. Across all relative densities (5%, 10%, and 15%), the experimentally measured weights were consistently lower than the nominal values derived from the CAD models. The effective weighted diameter (d_n,ew_) for each configuration was determined by iteratively modifying the strut diameter d_n_ within the CAD model until the computed weight matched the experimentally measured values (W_r_ = W_n_), resulting in a weight error of zero.

**Figure 9:**
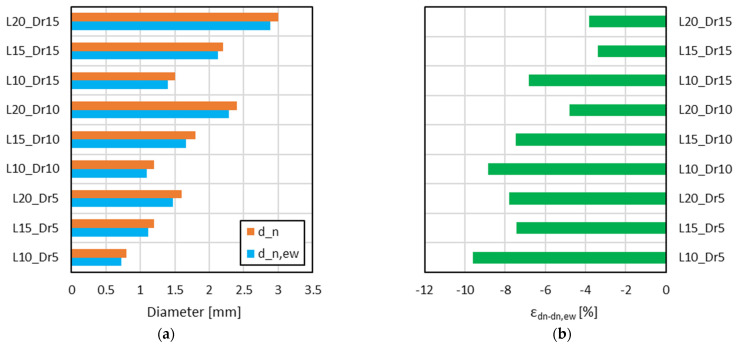
Comparison between d_n_ and d_n,ew_ (**a**) and relative error “ε_dn-dn,ew_” (**b**) for each lattice configuration.

The comparison between d_n_ and d_n,ew_ is illustrated in Figure 9a. The results indicated a systematic reduction in the effective strut diameter compared to the nominal input, with deviations generally decreasing as the cell dimension and relative density increased (Figure 9b). This trend suggests that larger cells and higher relative densities exhibit improved fabrication accuracy due to enhanced material deposition and better structural support during printing.

**Figure 10:**
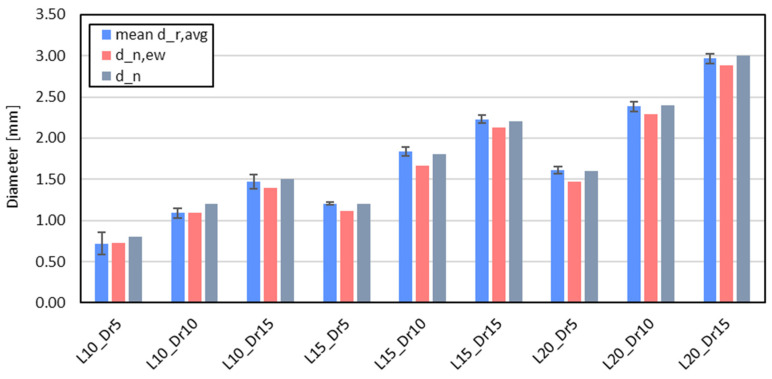
Comparison of mean strut diameter (mean d_r,avg_)—calculated across x, y, z, and s directions—against nominal strut diameter (d_n_) and effective weighted diameter (d_n,ew_), for each cell configuration.

Figure 10 provides a graphical comparison of three key diameters illustrated in this work: the nominal diameter (d_n_), the effective weighted diameter (d_n,ew_), and the averaged strut diameter (mean d_r,avg_). The mean d_r,avg_ reflects the actual strut diameter while accounting for any geometrical inconsistencies introduced by the printer. On the other hand, d_n,ew_ not only accounts for these geometrical inconsistencies but also incorporates the effect of voids present within the struts. Notably, d_n,ew_ can be considered as the ideal diameter—constant along the entire length and direction of the strut—that factors in both the printer’s geometrical inconsistencies and the voids within the structure. By comparing these two diameters (mean d_r,avg_ and d_n,ew_), the presence of voids in different configurations can be assessed. Furthermore, when both d_n,ew_ and mean_d_r,avg_ are compared to the nominal diameter (d_n_), it becomes evident that the printer consistently underestimates the true dimensions of the struts across all configurations analyzed. This comparison paves the way for the definition of an equivalent diameter, which will aid in understanding the mechanical response of the lattice structure.

All lattice configurations were fabricated using a direction-parallel (raster) pattern with the minimum body thickness. This pattern enables high infill density and precise material deposition in layers with regular polygonal geometries; however, it is susceptible to underfilled regions when applied to non-polygonal curvilinear structures [27]. In cell configurations with thin strut diameters, the limited cross-sectional dimensions restricted extrusion to the contour only, which effectively filled the strut’s interior due to its small size. As a result, these structures exhibited fewer voids, leading to a close agreement between the microscopy-measured and effective weighted diameters. However, the reduced strut size, combined with minimal support material during printing, increased geometric and dimensional inaccuracies relative to the nominal diameter. Conversely, for thicker strut diameters, the printing process allowed for the filling of the inner strut volume using the direction-parallel pattern. This, however, introduced a higher number of voids within the structure, resulting in greater discrepancies between the microscopy-measured and effective weighted diameters. Nonetheless, the microscopy-measured diameters remained closer to the nominal values, as the larger strut dimensions improved overall printing accuracy.

## 4. Conclusions

This study investigates the geometric and dimensional accuracy of ABS-based Cuboidal BCC lattice cells produced through a Fused Deposition Modeling process, addressing the challenges in fabrication, material deposition, and structural integrity.

The investigation centers on lattice structures with predefined geometries characterized by cell edge dimensions of 5, 10, 15, and 20 mm, and low relative densities of 5%, 10%, and 15%, where relative density is defined as the ratio of the lattice structure’s density to that of the solid material from which it is composed.

The results revealed that lattice cells with nominal dimensions of 5 mm presented critical issues, as their small size led to fragile struts that fractured during or immediately after the printing process. This highlighted the limitations of FDM in producing reliable lattice structures at smaller scales, which led to the exclusion of these cells from further analysis. Dimensional characterization showed that the printed lattice structures exhibited deviations from the nominal values ranging from 0.6% to 1.8%. These variations, however, did not correlate consistently with specific lattice parameters such as cell size, strut diameter, or relative density, suggesting that other process-induced factors contributed to the discrepancies. Further analysis of the strut geometry revealed that parallel-oriented struts achieved the highest dimensional/geometrical accuracy, with minimal deviation from the nominal values. The discrepancies observed in certain configurations, particularly L10_Dr5, were attributed to the optimization process of the slicing algorithm, which affected the deposition of material, leading to local material accumulation or insufficient deposition in some instances. The analysis also highlighted the influence of printing orientation on the overall dimensional/geometrical fidelity of the struts.

In addition to the dimensional and geometrical analysis, weight measurements provided valuable insight into the manufacturing consistency, revealing that the printed lattice cells consistently exhibited lower weight than the theoretical values derived from the CAD models. The observed weight discrepancies were primarily attributed to variations in material deposition and the presence of internal voids, which are characteristic of the FDM process. By iteratively adjusting the strut diameter to match the experimentally measured weight, it was possible to determine an effective weighted diameter for each configuration, which was generally smaller than the nominal values. This trend was more pronounced in smaller cell dimensions and lower relative densities, where limited extrusion capacity and insufficient support material resulted in greater deviations. Conversely, larger cells and higher relative densities exhibited improved fabrication accuracy, as larger strut sizes allowed for better material deposition and structural support.

From a practical perspective, this study provides a framework for assessing the manufacturability of lattice structures via FDM, offering quantitative insights into expected dimensional deviations. These findings are particularly relevant for industries such as aerospace, where precise control over geometrical/dimensional accuracy and material consistency is crucial for lightweight structural applications. Notably, the ability to fabricate lattice structures with controlled geometries and low relative densities, including those investigated in the present work with relative densities of 5, 10, and 15%, makes them strong candidates for use as core materials in sandwich structures, where weight reduction and energy absorption are critical design considerations. However, further optimization of the FDM process is necessary to improve the reliability of these structures for such applications. Future research should explore strategies to enhance print precision, including refined slicing algorithms, optimized extrusion parameters, and alternative support structures, to further advance the use of FDM in fabricating complex lightweight lattices for high-performance engineering applications.

While optical microscopy was utilized to assess external geometric features, its limitations in capturing internal microstructural details highlight the need for advanced imaging techniques. Future studies should incorporate scanning electron microscopy (SEM) to investigate filament arrangement, void distribution, and material consolidation in greater detail. Furthermore, analyzing the effect of printing direction on the geometrical and dimensional accuracy of the lattice structure would offer deeper insights. Additionally, mechanical performance testing of the produced lattice structures is essential for a more comprehensive evaluation of their suitability for load-bearing applications.

## Figures and Tables

**Table 1:** Manufacturer-specified process parameters for Stratasys F190 CR printer, optimized for layer adhesion and residual stress minimization.

Parameter	Value
Chamber Temperature [°C]	75
Nozzle ABS temperature [°C]	190
Nozzle support temperature [°C]	265
Nozzle diameter [mm]	0.400
Slicing height [mm]	0.127

**Table 2:** Experimental configurations for Cuboidal BCC lattice structures, including cell lattice dimensions, relative densities, cross-section strut diameters, and nominal cell weights.

Cell Configuration, LN_DrM	Cross-Section Strut Diameter, d_n_ [mm]	Nominal Cell Weight, W_n_ [g] *
L5_Dr5	0.4	0.0074
L10_Dr5	0.8	0.0945
L15_Dr5	1.2	0.3189
L20_Dr5	1.6	0.7558
L5_Dr10	0.6	0.0258
L10_Dr10	1.2	0.2065
L15_Dr10	1.8	0.6968
L20_Dr10	2.4	1.6516
L5_Dr15	0.8	0.0346
L10_Dr15	1.5	0.3155
L15_Dr15	1.9	1.0203
L20_Dr15	3.0	2.5233

* The nominal cell weight was derived from the CAD model, based on the density of ABS material (1.05 g/cm^3^) as provided by the manufacturer.

**Table 3:** Comparison of mean strut diameter (mean d_r,avg_)—calculated across x, y, z, and s directions—against nominal strut diameter (d_n_), for each cell configuration.

Cell Configuration, LN_DrM	Nominal Strut Diameter, d_n_ [mm]	Mean Strut Diameter, Mean d_r,avg_ [mm]
L10_Dr5	0.8	0.72 ± 0.14
L15_Dr5	1.2	1.20 ± 0.07
L20_Dr5	1.6	1.61 ± 0.04
L10_Dr10	1.2	1.09 ± 0.06
L15_Dr10	1.8	1.84 ± 0.06
L20_Dr10	2.4	2.38 ± 0.06
L10_Dr15	1.5	1.47 ± 0.09
L15_Dr15	2.2	2.23 ± 0.05
L20_Dr15	3.0	2.97 ± 0.06

## Data Availability

Data are contained within the article.

## Abbreviations

The following abbreviations are used in this manuscript:

N	Cell edge dimension [mm]
M	Relative density percentage [%]
LN_DrM	Lattice configuration notation [-]
ρ_lattice_	Density of the lattice [g/cm^3^]
ρ_solid_	Density of ABS material [g/cm^3^]
x, y	Directions parallel to printing orientation [mm]
z	Direction orthogonal to printing orientation [mm]
s	Direction for inclined struts [mm]
d_n_	Nominal diameter from CAD model [mm]
d_r,avg_	Averaged strut diameter [mm]
d_n,ew_	Effective weighted diameter
mean d_r,avg_	Mean strut diameter
W_r_	Measured weight of printed samples [g]
W_n_	Nominal weight from CAD model [g]
ε_Wn-Wr_	Weight relative error [%]
ε_dn-dn,ew_	Relative error between nominal and effective weighted diameter [%]
δ	Cell dimension deviation [%]
